# The right to mental integrity in the age of neurotechnology: constructing scope and exploring permissible limitations

**DOI:** 10.1093/jlb/lsaf010

**Published:** 2025-06-03

**Authors:** Sjors Ligthart

**Affiliations:** Tilburg Law School, Tilburg University, Tilburg University, Warandelaan 2, 5037 AB, Tilburg, The Netherlands; Utrecht University, School of Law, Johanna Hudiggebouw, Achter Sint Pieter 200, 3512 HT Utrecht, The Netherlands

**Keywords:** mental integrity, human rights, neurotechnology

## Abstract

One way to ensure adequate legal protection against existing and emerging forms of mental interference is by specifying the human right to mental integrity. This paper considers three possible constructions of the scope of this right in human rights law. It argues that the Mental Control View and the Direct Harmful Interference View fall short of providing a persuasive definition of the right. Rather, it is proposed to construct the scope of the right along the lines of the Significant Mental Interference View. Meanwhile, the *directness* of a mental interference and the psychological *harm* it entails are plausibly relevant factors to the potential justification of rights infringements.

## I. INTRODUCTION

Since recent years, academics, ethics committees, and policymakers are increasingly considering the legal regulation of emerging neurotechnologies that enable the direct alteration of neural correlates of mental states and processes.^1^ This includes discussions on how human rights should protect against unsolicited interferences with people’s brains and minds.^2^ In a recent report on the impact, opportunities, and challenges of neurotechnology in relation to human rights, the Human Rights Council Advisory Committee concluded that ‘[n]eurotechnologies affect human rights in a unique manner’ and that developing ‘an actionable human rights approach is of the utmost importance’.^3^

Scholars have proposed different ways of guaranteeing adequate legal protection in this area, including by the recognition of new human rights; by reshaping existing rights; or by reaffirming and strengthening established human rights through evolutive interpretation.^4^ The report by the Advisory Committee leans toward the latter.^5^

Although these proposals differ in *how* adequate human rights protection of the mind should be realized,^6^ they generally agree upon the personal interests that deserve protection. One personal interest central to the present debate is our interest in *mental integrity*.^7^ For example, Ienca and Andorno have argued for a specific human right to mental integrity that should protect against unsolicited harmful interference with people’s minds by using neurotechnology.^8^ Bublitz, too, puts emphasis on the right to mental integrity, as part of the protection of personal integrity inherent in established human rights, which would protect against substantive alterations of mental states and processes, including by neurointerventions.^9^ In recent reports on human rights and neurotechnology, such as for the UN Human Rights Council and the Council of Europe, safeguarding persons’ mental integrity through human rights law is a central theme.^10^

Meanwhile, whereas a right to *bodily* integrity is clearly carved out in the law and is regularly applied in individual cases, case law on a right to *mental* integrity is still scarce (though not completely lacking). As a consequence, many aspects of the right to mental integrity are still unclear, including its scope and the permissible limitations the right allows for.^11^ For example, is the infliction of mental harm or psychological distress required to infringe the right? To what extent does the right apply to indirect means of altering mental states, such as through subliminal advertising or persuasive technologies? And under which circumstances may the right to mental integrity be outbalanced by competing public or private interests?

Guaranteeing adequate, effective, and actionable human rights protection of mental integrity—also in relation to neurotechnology and other emerging techniques that increasingly enable mental interference by third parties^12^—requires specifying this right. In their recent report, the Advisory Committee of the Human Rights Council recommended that the United Nations treaty bodies should draft new general comments to clarify and strengthen the human rights protection of the inner mental space: the right to mental (and bodily) integrity ‘should be considered a priority’.^13^

This paper aims to contribute to clarifying our understanding of the human right to mental integrity, by considering the right’s scope and its permissible limitations. It approaches the right to mental integrity as an existing right, enshrined in the established framework of human rights law.^14^ This approach is briefly explained in Section II, followed by an analysis of the right’s scope and its permissible limitations in Sections III and IV. Section V draws conclusions.

Before proceeding, the following points are in order. First, this paper focuses on the negative understanding of mental integrity: as a right against certain forms of mental interference.^15^ While acknowledging the relevance of positive freedoms over the mind, such as cognitive liberty,^16^ a discussion of those positive freedoms is beyond the scope of this paper.^17^

Second, the right to mental integrity may sometimes overlap with the right to bodily integrity. Think, for example, of modulating *mental* processes through *physical* interventions, like psychopharmaceuticals and neurotechnological brain stimulation.^18^ The permissibility of such interventions, one may argue, can be dealt with under the well-established right to bodily integrity. However, the effects of such interventions at the bodily level may often be minor (especially when the intervention is non-invasive) and ‘may be seen as insufficient to trigger human rights protection’.^19^ Rather, as different authors have pointed out: what really matters are the *mental effects* of such interventions, which are primarily covered by the right to mental integrity.^20^ Therefore, this paper focuses on the right to mental integrity (though without excluding the potential relevance of the right to bodily integrity in at least some cases).

Finally, this paper considers the right to mental integrity as protected by the International Covenant on Civil and Political Rights (ICCPR) and the European Convention on Human Rights (ECHR). Meanwhile, some parts of the analysis may equally apply to other human rights instruments.

## II. LEGAL FOUNDATIONS OF THE RIGHT TO MENTAL INTEGRITY

Neither the ICCPR nor the ECHR explicitly recognizes a right to mental integrity. They omit reference to the right in contrast to other human rights instruments. For example, Article 5(1) of the American Convention on Human Rights prescribes that ‘Every person has the right to have his physical, mental, and moral integrity respected’. Likewise, Article 3(1) of the European Charter of Fundamental Rights prescribes that ‘Everyone has the right to respect for his or her physical and mental integrity.’ Furthermore, Article 17 of the Convention on the Rights of Persons with Disabilities provides that ‘Every person with disabilities has a right to respect for his or her physical and mental integrity on an equal basis with others’.

In the ICCPR and ECHR, meanwhile, the protection of mental integrity is inherent in other human rights. First, grave interferences with mental integrity can violate the prohibition of torture, inhuman and degrading treatment, and punishment.^21^ According to Nowak/Schabas, the prohibition of ill-treatment of Article 7 ICCPR contains ‘the right to physical and spiritual integrity’.^22^ According to General Comment No. 35, Article 7 ICCPR aims ‘to protect both the dignity and the physical and mental integrity of the individual’. A similar right against (less severe) mental injury is inherent in the right to security of person, laid down in Article 9 ICCPR, which ‘concerns freedom from injury to the body and the mind, or bodily and mental integrity’.^23^

Furthermore, putting less emphasis on the protection from (severe) mental injury, a right to mental integrity is also inherent in the umbrella rights to privacy (Article 17 ICCPR) and respect for private life (Article 8 ECHR) – both covering the protection of ‘personal integrity’,^24^ which is typically taken to consist of both bodily and mental integrity. This follows most clearly from the case law of the European Court of Human Rights (ECtHR), considering that the right to respect for private life pursuant to Article 8 ECHR ‘provides for the protection of physical and mental integrity’.^25^ This right to mental integrity, also referred to as a right to ‘psychological’ or ‘moral’ integrity (which the ECtHR uses interchangeably),^26^ is associated with the protection of mental health.^27^ But its protective scope appears broader, as it also pertains to bullying at school,^28^ well-founded fear of physical abuse,^29^ and loss of honor and reputation, for example, by others publishing defamatory statements in the media.^30^

One guiding principle for the applicability of the right to mental integrity inherent in Article 8 ECHR is that, for infringing the right, an interference should have ‘sufficiently adverse’ effects.^31^ As De Vries explains, ‘Article 8 only applies when a person’s physical or psychological integrity is adversely affected to a certain degree’.^32^ Although this general threshold provides some direction, it is still vague and has not been further developed in the case law yet.^33^ What does the ECtHR mean by ‘adverse’ effects? Would, for example, making people happier, more attentive, or less aggressive qualify as such? And when are the effects of an interference ‘sufficiently’ adverse so as to infringe the right to personal integrity?

Regarding the right to *bodily* integrity, many interferences appear to meet the sufficiently adverse threshold: ‘even a minor interference with the physical integrity of an individual must be regarded as an interference with the right to respect for private life under Article 8 if it is carried out against the individual’s will’.^34^ Taking a buccal swab without valid consent, for instance, infringes the right to bodily integrity under Article 8 ECHR; even though it ‘is an act of a very short duration, it usually causes no bodily injury or any physical or mental suffering, and thus is of minor importance’.^35^ Furthermore, regarding medical treatment, Harris et al. write that without consent, such treatment raises serious issues within the scope of private life, ‘however slight the intervention’.^36^ Apparently, ‘slight’ interferences with the body that are ‘of minor importance’ can still qualify as ‘sufficient’ and thus infringe the right to bodily integrity.

Should a similar approach apply to the right to mental integrity? And what would such mean for the right’s protective scope? Let’s consider these questions in more detail below.

## III. CONSTRUCTING THE RIGHT’S SCOPE

Whereas the right to mental integrity has received relatively little attention in human rights analyses so far,^37^ in the philosophical literature, constructing the right is a central topic in discussions over the normative implications of *moral* rights vis-à-vis emerging neurotechnologies.^38^ Even though human rights are often considered to reflect moral guarantees, a moral right to mental integrity need not straightforwardly imply or correspond to its legal counterpart. Still, when aiming to clarify and construct the meaning and scope of a human right to mental integrity, the philosophical literature could provide useful guidance.^39^

Drawing from both the legal and philosophical literature, this section will distinguish and discuss three general understandings of a right to mental integrity and consider their potential fit within the established framework of international and European human rights.^40^ For this, general principles on the interpretation of human rights should be taken into account, including: human rights should be interpreted so as to realize the object and purpose of human rights treaties, not to restrict them; human rights should be practical and effective, not theoretical and illusory; and interpretations of different treaty provisions should seek internal consistency. The three different understandings of the right to mental integrity that will be consecutively discussed, will be referred to as (i) the Mental Control View, (ii) the Direct Harmful Interference View, and (iii) the Significant Mental Interference View.

### III.A. The Mental Control View

A first way of constructing the right to mental integrity is by holding that every person has the liberty to *control* what happens to their own mind and the neural parameters of mental states and processes. For example, Lavazza defines mental integrity as ‘the individual’s *mastery* of his mental states’.^41^ Likewise, Hildt holds that the ‘right to mental integrity stresses a person’s right to *control* their brain states’.^42^ In their draft report, the Human Rights Council Advisory Committee wrote that ‘mental integrity would ensure individual’s *control* over body and mind providing the basis to prohibit unsolicited exposure to neuromodulation procedures, even in medical context’.^43^ In their final report, the Committee mentions that neurotechnologies could violate the right to ‘personal integrity (…) when endangering individuals’ *autonomous control* over their body and mind’.^44^

Such understandings of a right to mental integrity resemble some aspects of the conceptualization of a right to ‘mental self-determination’ as defended by Bublitz and Merkel, of which the principal premise is that everyone has a right to control their own state of mind.^45^ In the negative dimension, the right to mental self-determination protects from severe mental interference by others: everyone should ‘refrain from interventions severely interfering with another’s mental integrity by undermining mental control or exploiting pre-existing mental weaknesses’.^46^

Bublitz and Merkel ground the right to mental self-determination in the idea of self-ownership over our minds: ‘[i]n the wake of Locke, libertarians believe that persons have property rights in their body; persons literally own (the physical part of) themselves. Ownership discussions focus on the relation of persons to their bodies, their liberty (e.g. vis-a-vis slavery) and the fruits of their labor. But what is even more constitutive of a subject than her body is her mind. So, whoever grants self-ownership of persons over their bodies has a compelling reason to concede self-ownership over minds’.^47^ Douglas and Forsberg likewise argue that ‘we might think that considerations of self-ownership and personal sovereignty in fact provide stronger support to a moral right to mental integrity than to a moral right to bodily integrity’.^48^

From this perspective, our interest in self-ownership and control could indeed be a plausible moral *justification* for recognizing a right to mental integrity.^49^ However, referring to these justifications will not yet clarify much about the right’s *scope*. For example, which types of interference would infringe a right to the individual’s ‘mastery of his mental states’? When will a person’s right to ‘control their brain states’ be infringed? What types of mental influence will qualify as a control-undermining interference in this regard, and which mental states should be relevant?

As Zohny et al. write, there are numerous possible interpretations of the idea of being deprived of ‘full control’ over one’s own mental states or of controlling one’s own consciousness.^50^ One such interpretation, which they consider least plausible, is that we usually have the ability to organize and deliberately select our mental states, such as thoughts, and the broader contents of our consciousness. However, they argue, this seems an incorrect description of how mental states arise from a subjective standpoint. We do not consciously select our thoughts, desires, and beliefs: they usually just arise unbidden in the mind.^51^ In the same vein, Bublitz and Merkel highlight that our factual powers to control our minds are much more limited than we may often assume: we cannot concentrate as we wish, remember all we may want, nor change our beliefs just by wanting them to be different.^52^

Accordingly, Zohny et al. assume that for the interpretation of mental integrity, any reference to depriving individuals of their ‘mastery’ or ‘full control’ over mental states and consciousness cannot pertain to mental control in an absolute sense, ‘because this is the kind of control that we lack anyway’.^53^ Rather, they argue, reference to control in recent understandings of mental integrity plausibly relates to the more modest interpretation of ‘being free from others *interfering* with one’s thoughts or mental states’.^54^ Such an understanding of the right to mental integrity has been defended by others, too, both philosophers and lawyers,^55^ with some of them explicitly referring to a ‘right against mental interference’.^56^

Meanwhile, constructing the right to mental integrity as a right against mental interference still leaves us with many open questions about the right’s scope. For example, what types of ‘mental interference’ would count as a rights infringement in this understanding? And what kinds of mental phenomena does the right aim to protect from interfering with? The following sections discuss two general proposals that aim to specify the right to mental integrity as a right against *certain* forms of mental interference.

### III.B. The Direct Harmful Interference View

In their oft-cited paper on human rights in the age of neuroscience and neurotechnology, Ienca and Andorno propose a reconceptualization of the existing human right to mental integrity. This right, they argue, should provide specific normative protection from unauthorized alterations of persons’ neural computation, which potentially result in direct harm.^57^ More specifically, they submit:

For an action X, to qualify as a threat to mental integrity, it has to: (i) involve the direct access to and manipulation of neural signalling (ii) be unauthorized – i.e. must occur in absence of the informed consent of the signal generator, (iii) result in physical and/or psychological harm.^58^

To infringe the right to mental integrity on this account, a non-consensual mental interference should operate *directly* through the brain and must result in physical and/or psychological *harm*.

This construction of the right is clearly more specific compared to a right ‘to control’ one’s own mental states or a right ‘against mental interference’. But it also raises novel questions—in particular regarding the precise meaning and normative significance of the two central conditions of the proposed reconceptualization: ‘directness’ and ‘harm’. Apart from providing some examples of potential threats to mental integrity—including malicious brain-hacking, deep brain stimulation in medicine, soldier enhancement with BCI, and invasive brain-washing interventions—Ienca and Andorno do not further elaborate upon what, exactly, the requirements of directness and harm consist of, nor why they are compelling conditions for the delimitation of the right’s scope. Why should the right to mental integrity only protect from direct interferences but not from *indirect* alterations of mental states?^59^ Against what types of physical or psychological ‘harm’ would the right protect? And why should the infliction of physical and/or psychological harm be a condition for the right’s applicability in the first place?^60^ Let’s consider these questions in more detail below.

#### 1. The Requirements of ‘Directness’

Let’s start with the requirement of ‘directness’, which refers to the *manner* of causing mental effects in others. There has been discussion over the normative significance of direct vs. indirect interventions in people’s mental states. Bublitz and Merkel have described these two different ways of mental interference as follows:

Tentatively, indirect (or external) interventions are those stimuli which are perceived sensually (i.e. heard, seen, smelled, felt, even if not apprehended or reflected upon consciously) and pass through the mind of the person, being processed by a host of psychological mechanisms. Thus, conscious communication in all its forms is an indirect intervention. By contrast, direct (or internal) interventions are stimuli reaching the brain by other routes than sensual perception. The main difference is that direct interventions can be primarily understood as electro-chemical or physical reactions following the laws of nature whereas indirect interventions involve psychological laws (or dynamics) and relate to what is being perceived.^61^

Arguments over the normative significance of this distinction typically appeal to the *level of control* people have over either direct or indirect interventions. The general assumption is that people have the greatest control over *indirect*, sensory interventions that are *consciously perceived*. Such interventions give people the opportunity to think about what they see, hear, or smell; reflect upon those stimuli; and process them. This process would create the possibility to decide how one integrates the perceived information into one’s own psychological framework, for example, by deliberating, questioning or challenging the information before it indirectly shapes a person’s beliefs, desires, or traits.^62^

The level of control over indirect interventions is considered to be reduced when stimuli are *subconsciously* processed, such as subliminal messages in advertisements or political campaigns. At the most extreme end of the spectrum, non-sensory *direct* interventions are considered to *bypass* any psychological process. They operate via purely physical–biological processes in the brain, such as psychopharmaceuticals and brain stimulation, over which the level of conscious control people have is typically low (if any).^63^

These distinctions in levels of *control* are, in turn, considered normatively relevant in relation to rights that aim to protect the *autonomy* over mental states and processes, such as the right to mental integrity.^64^ For example, according to Ratoff, the moral ‘right to mental autonomy (…) is your right to form attitudes in light of overt reasons – that is, in light of reasons that have their influence on your thinking without circumnavigating your consciousness or awareness’.^65^ Considering the moral right to mental integrity, Zohny et al. argue that the threat to mental integrity posed by some neurotechnologies ‘is that they simulate the nervous system in a way that side-steps the opportunity to rationally evaluate their potential influence on mental states or traits, and thereby to control the degree to which they do in fact influence (or interfere with) them’.^66^ From a legal point of view, Bublitz has argued that:

the scope of the right against mind-interventions has to be confined to interferences that undermine mental self-determination to a degree that fails a test of what is reasonable in a highly cooperative, interactive, and communicative society. And this, I claim, is true of interventions that bypass mental control. (…) In light of this premise, the relevance of mental control and integrity – and the direct versus indirect distinction – becomes evident. The more control, the better.^67^

In view of a ‘right against unwanted mind-interventions’, Bublitz considers interventions that undermine or bypass mental control ‘particularly troublesome’.^68^

Note that not everyone agrees upon the moral significance of this direct–indirect distinction. For example, considering both neural and environmental modulation of mental states, Douglas has challenged the idea that non-perceptual interventions are more objectionable in terms of the mental interference they involve than perceptual interventions.^69^ In the same vein, Levy holds that indirect interventions also often produce mental effects that bypass a person’s psychological capacity. Moreover, he argues that, generally, indirect interventions are responsible for far more injustice, harm, and inequality than direct interventions.^70^ Referring to the work of Daniel Kahneman, Ryberg likewise maintains that the view that indirect interventions are controllable ‘is of course sometimes true, but quite often it is not’.^71^

The present paper does not take a position in the debate on the moral significance of direct vs. indirect mental interference. Rather, for the sake of argument, let’s assume that, in general, direct interventions, such as neurotechnological brain stimulation, leave less space for mental *control* than indirect interventions, such as influencing mental states through consciously perceived perceptual stimuli. Let’s also assume that the extent to which a mental interference restricts or bypasses mental control is relevant to human rights that protect the *autonomy* over mental states and processes, including the right to mental integrity. The question that arises, then, is *how exactly* could this direct–indirect distinction add to constructing and applying the right to mental integrity? Is it a compelling factor for delimitating the right’s scope, as proposed by Ienca and Andorno? Or would the directness of a mental interference—and the accompanied decrease or circumvention of mental control—rather be a relevant consideration to the question of whether an interference can, potentially, be justified?

From the perspective of positive human rights law, constructing the scope of the right to mental integrity along the lines of the direct–indirect distinction seems not persuasive, as it will plausibly curb existing human rights protection of mental integrity, rather than strengthening it. This is because, under both the ECHR and ICCPR, inducing psychological effects in others through *indirect* means can equally infringe the right to mental integrity. For instance, causing well-founded fear by oral threats of violence or by harassment of a person’s close relatives—clearly not qualifying as direct interventions—may sometimes infringe the right to mental integrity under Article 8 ECHR.^72^ Furthermore, in a case on publishing defamatory statements and sexual photographs of a person in the media, the ECtHR held that this had ‘seriously prejudiced the applicant’s honour and reputation and was harmful to his psychological integrity and private life’.^73^ Regarding Article 17 ICCPR, Nowak/Schabas even mention intentional ‘trivial insults’ by executive organs as potential infringements of personal integrity.^74^ Furthermore, regarding Article 3 of the EU Charter on Fundamental Rights, the EU Network of Independent Experts on Fundamental Rights notes that the right to mental (and bodily) integrity ‘is a fairly broad right’, applying to a broad range of serious and less serious forms of interference with a person’s body and mind, including by ‘strong noise’ and ‘brainwashing’.^75^ As these examples illustrate, (potential) infringements of the right to mental integrity are currently not limited, in principle, to direct (electro-chemical or physical) interferences with mental states and processes. Constructing the right to mental integrity as a right that is *only* applicable to direct interventions would lead to a more limited right than it is today, which speaks against adopting this requirement.

Note, finally, that the direct–indirect distinction neither appears a necessary condition for the applicability of other human rights that protect mental autonomy, such as the right to freedom of thought.^76^ Various *indirect* means of influencing mental states are often even referred to as paradigmatic violations of this right, including indoctrination, brainwashing, and re-education camps.^77^ Moreover, the Special Rapporteur on Freedom of Religion or Belief recently highlighted that a ‘growing body of legal scholarship supports the claim that freedom of thought includes freedom from manipulation. While modification bypasses psychological processes to directly alter biological function, manipulation engages and controls psychological processes’.^78^ In other words: next to directly interfering with a person’s thoughts, indirect interventions into their psychological processes fall within the scope of the right to freedom of thought too.^79^ Promoting internal consistency and harmony between different treaty provisions would thus provide another argument against accepting the directness-requirement for the applicability of the right to mental integrity.

In sum, adopting the directness-requirement to construct the scope of the right to mental integrity will plausibly restrict existing human rights protection of the human mind. It will confine the scope of this right to mental changes induced by psychopharmaceutical, neurotechnological, and some other physical interventions,^80^ compromising existing human rights protection against a variety of indirect mental interferences. Limiting the right’s scope along these lines would oppose settled case law on the right, such as on defamation and threats of violence, and would be inconsistent with the interpretation of other rights that protect the interest in mental autonomy, like the right to freedom of thought. Curbing the protective scope of the right to mental integrity in this way seems at odds with the principle that human rights should be interpreted in a way that is practical and effective and to realize the object and purpose of human rights treaties, not to restrict them. Therefore, the direct nature of a mental interferences should not be adopted as a necessary requirement for infringing the right to mental integrity. It may, however, be a relevant consideration to determining the right’s permissible limitations, which will be discussed in Section IV.

#### 2. The Requirement of ‘Harm’

Let’s turn to the other central requirement of Ienca and Andorno’s proposal: for infringing the right to mental integrity, an interference must result in either physical or psychological *harm*. What types or levels of harm the authors think should be required for infringing the right remain unclear. Without further specification, such a general harm requirement will not add much to specifying the scope of the right. As Zohny et al. pointed out, what counts as being harmed or made worse of in this regard is hard to settle.^81^ For example, ‘it cannot be that the concern over mental integrity comes down to being protected from *any* harmful mental interference. People interfere with our mental states all the time in ways that harm us on any plausible sense of that term, and in ways no one thinks we have a moral or legal right to be protected from.’^82^ Zohny et al. consider the example of being sincerely told by a loved one that you are a disappointment: although such a notification plausibly interferes with mental states in devastating ways, it does not follow that a (moral) right to mental integrity protects against others potentially telling us things like these.^83^

Furthermore, depending on how one defines harm, the harm requirement could also *exclude* certain mental interferences from the right’s scope that would, intuitively, qualify as paradigmatic infringements. Think, for example, of making people happier, more emphatic, or less aggressive by secretly administering small electric currents to certain brain areas. Such interventions, which some may consider paradigmatic infringements of the right to mental integrity,^84^ may not be covered by the right if either physical or psychological harm is required.^85^

Moreover, from a legal point of view, constructing the right to mental integrity as a right that *only* protects against interferences that cause physical or psychological harm seems conceptually narrower than current understandings of the right. Indeed, the *absolute* protection of mental integrity as part of the prohibition of torture and ill-treatment only applies in cases of severe physical or mental suffering.^86^ However, harm does not appear to be required for the application of the *qualified* right to mental integrity, at least not as far as this right is inherent in Article 8 ECHR. For instance, according to Bublitz, the right to mental integrity in the meaning of Article 8 ECHR seems conceptually broader, capturing interferences that do not amount to setbacks to mental health, like in cases of prejudiced honor and reputation, such as through defamation.^87^ Likewise, Biber and Capasso emphasize that ‘the ECtHR has broadly interpreted the terms mental and psychological integrity. According to the case-law, these terms do not cover only the setbacks in mental capabilities but also situations in which no clinical-pathological mental disorders occur’.^88^

In sum, without further specification, the harm-requirement will add little (if anything) to our understanding and delineation of the protective scope of a right to mental integrity in human rights law. On the one hand, many mental effects that arise from social interaction can and will cause different levels of mental harm, including by being bullied, laughed at, or misunderstood. We do not plausibly have a legal right to be protected from all such harmful mental interferences (while some may be relevant, such as bullying). As such, the scope of a right against ‘harmful mental interference’ may be too broad. Meanwhile, on the other hand, adopting the harm-requirement may also exclude certain paradigmatic infringements of the right to mental integrity from its scope, such as non-consensual non-invasive brain stimulation to increase a person’s empathy or happiness. From this perspective, a right against harmful mental interference may be considered too narrow a right—while the infliction of harm seems no necessary condition for infringing the right to mental integrity in established human rights law.

Therefore, without further specification, the infliction of physical or psychological harm appears to be a non-persuasive requirement for delimitating the scope of the right to mental integrity. This is, however, not to say that *if* a mental interference clearly causes psychological harm, for example, by causing anxiety or distress, such would be legally irrelevant. Similar to the directness of a mental interference, its harmful effects may be a relevant consideration when determining the right’s permissible limitations (Section IV).

In conclusion, neither the requirement of ‘direct’ interference nor of physical and/or psychological ‘harm’ appear compelling for constructing the scope of the human right to mental integrity. The legal significance of these requirements is largely unclear, and the current interpretation of the right is conceptually broader. Therefore, categorically excluding all indirect and harmless mental alterations from the right’s scope is unwarranted. It will plausibly restrict rather than strengthen human rights protection against non-consensual interventions into people’s minds.

But what if we reject both harm and directness as central requirements for infringing the right to mental integrity? Wouldn’t this lead to an overinclusive understanding of the right? Without delimitation, the right will apply to almost every trivial and non-trivial mental interference by others. These may include a broad range of everyday, undoubtedly permissible social interactions that affect other people’s minds, for example, by causing anxiety, disappointment, or anger, such as through the news; advertisements; and interactions with friends, family, and strangers.^89^ To prevent the right from being overinclusive, some kind of threshold should plausibly apply. This brings us to a third possible construction of the right’s scope: the Significant Mental Interference View.

### III.C. The Significant Mental Interference View

Douglas and Forsberg understand a legal right to mental integrity as, generally speaking, ‘a right against (certain kinds of) nonconsensual interference with the mind’.^90^ Analogous to the right to bodily integrity, they think it plausible that some types of non-consensual mental influence will and should not infringe this right, because the effects are *not significant* enough:

If I wave my hand near your arm, causing the hairs on your arm to quiver, I have not infringed your right to bodily integrity, even if I do this without your consent; the effect of the influence is not significant enough. Similarly, there may be mental influences that fail to infringe the right to mental integrity because their mental impact is too insignificant.^91^

Exactly how significant a mental influence must be to infringe the right to mental integrity, Douglas and Forsberg set aside for further investigation. Elsewhere, Douglas has suggested that a mental interference might be too trivial to infringe the right in virtue of ‘falling below some threshold of magnitude’.^92^ In the same vein, Bublitz has proposed—also in analogy with bodily integrity—to define interferences with mental integrity as ‘actions that detrimentally affect the mind, i.e., mental states, processes, functions, and abilities, above a certain threshold of seriousness’.^93^ This includes the infliction of pain and mental injury, causing detriment to mental health, and other undesirable, non-trivial changes to the mind. More specifically, in Bublitz’s view, the right to mental integrity applies to ‘[s]ubstantive alterations of mental functioning, capacities, or important mental states’,^94^ including ‘non-consensual “improvements” of mental functioning, such as coercive treatments in forensic or psychiatric settings, or coercive cognitive enhancement’.^95^

The scope of such a right against *significant* mental interference is plausibly broader compared to a right against *direct harmful* mental interference (discussed above). Meanwhile, adopting a threshold of ‘seriousness’ or ‘significance’ could prevent the right from being overinclusive. For instance, in both Douglas’s and Bublitz’s interpretation, trivial mental effects fall outside the right’s scope.^96^ Such a construction of the human right to mental integrity seems persuasive, also because it aligns with how the ECtHR understands the right to personal integrity under Article 8 ECHR. As previously discussed, generally, Article 8 ECHR applies when the effects of an interference on the person’s physical or mental integrity are ‘sufficiently adverse’.^97^ What exactly this threshold consists of is yet unclear.^98^ But as discussed in Section II, for interferences with bodily integrity, the ‘sufficiently adverse’ threshold neither appears strict nor very demanding, as even ‘slight’ bodily interferences ‘of minor importance’ can qualify as ‘sufficient’ and infringe the right to bodily integrity.

This may feed the objection that constructing the right to mental integrity as a right against ‘significant’ mental interference—that is, interferences above a threshold of seriousness—is unpersuasive, because such a threshold (i) is unclear and ambiguous and (ii) bears the risk of being too low, possibly bringing into the right’s scope all kinds of serious, yet undoubtedly permissible, mundane mental influences inherent in social interaction.^99^ From a legal point of view, however, this objection is not compelling.

First, using unspecified and open norms and thresholds is a typical feature of the law—human rights law in particular. For instance, regarding the protection of ‘private life’ within the meaning of Article 8 ECHR, the ECtHR holds that ‘[t]he concept of “private life” is a broad term not susceptible to exhaustive definition’.^100^ Likewise, regarding the prohibition of ‘torture’, ‘inhuman’ and ‘degrading’ treatment of Article 7 ICCPR, the Human Rights Committee does not think it necessary to further specify these terms by drawing up a list of prohibited acts or by establishing sharp distinctions between the different forms of prohibited treatment.^101^ Such open and unspecified concepts appear not to be problematic for interpreting, applying, and developing human rights on a case-by-case basis. On the contrary, they enable the dynamic and evolutive interpretation and formation of rights and freedoms that are central to human rights law.

The same applies *mutatis mutandis* to the use of generally formulated thresholds for determining rights infringements, such as the ‘minimum level of severity’ threshold of the prohibition of ill-treatment and the threshold of ‘sufficiently adverse’ effects for infringing the right to personal integrity under Article 8 ECHR. Due to their formulation in general terms, these thresholds can adapt to ongoing developments in society. For example, regarding Article 3 ECHR, Harris et al. observe that over time, in practice, ‘the “threshold” has been lowered to cover certain intermediate forms of ill-treatment (…) that would not have been in the minds of the Convention drafters’.^102^ Hence, the objection that delimitating the right to mental integrity through a general threshold, such as requiring ‘significant’ interference that attains a certain level of ‘seriousness’, is unconvincing *because* it is vague and unspecified, is, from a legal point of view, unpersuasive.

Still, an opponent of the Significant Mental Interference View could argue that a general and unspecified threshold of ‘significance’ or ‘seriousness’ could lead to an overinterpretation of the right’s scope, applying to an extremely broad range of both exceptional and everyday non-trivial mental influences, including those stemming from the news, advertisement, education, and interpersonal relations and conversations. But again, from a legal perspective, this objection is not convincing, for at least three reasons.

First, human rights typically apply in the vertical relation between States and citizens. Generally, they do not address issues between private actors.^103^ Hence, whereas everyday social interactions and mental influences between citizens may possibly infringe their *moral* rights, they will not, typically, infringe human rights like the right to mental integrity.

Second, even if we assume (some) horizontal effect, then, still, only *non-consensual* interferences will be capable of infringing people’s right to mental integrity. Much of the mental influences we expose ourselves to through our social lives, including the news, advertisement, and conversations with others, are influences of which we are generally aware and hence to which we are capable to consent (unlike influences that we are unaware of, which operate below the level of conscious awareness). In fact, to many mundane mental influences we do, indeed, somehow consent, either implicitly or explicitly.^104^ In choosing to read the news, visiting a crowdy pub, and continue listening to others’ arguments, for example, we at least implicitly consent—ie through our willful actions—to being influenced by the information, impressions, views, and ideas to which we expose ourselves. Therefore, it is implausible to hold that all, or even most, of the mundane interferences with our minds to which we are exposed would infringe the right to mental integrity if the right’s scope is only delimited by a general threshold of ‘significance’ or ‘seriousness’.^105^ Many such interferences will not infringe the right, because the right-holder can often be considered having granted, either explicitly or implicitly, consent to them (and, thus, waived their right to mental integrity in the specific circumstances).

Third, even if we assume that a general threshold of ‘significance’ or ‘seriousness’ would be undemanding and, therefore, would imply a broad scope of the right to mental integrity, such will not yet mean that all kinds of *prima facie* permissible acts and mental influences will therefore become prohibited. A broad *scope* of the qualified right to mental integrity need not imply overprotection by the right. In human rights law, the scope of qualified rights is typically broad. For example, Article 8 ECHR protects a person’s sphere of privacy and personal autonomy, the scope of which ‘has expanded substantially over the years as the Court has interpreted the notion of private life to cover a broad range of interests’.^106^ Likewise, constitutional rights in national jurisdictions are often formulated in broad and general terms. They typically protect many different aspects of people’s private and public lives. For example, the right to freely develop one’s personality pursuant to Article 2(1) of the German Basic Law includes—among many other things—the right to feed pigeons in the park and to go riding in the woods.^107^

However, a broad scope of human rights does not yet imply that every interference with the protected interest also *violates* the right and will therefore be *prohibited*. Most human rights, including the rights to privacy and respect for private life (Articles 17 ICCPR, 8 ECHR), are qualified rights. They have broad scopes but allow for a range of exceptions and limitations. Generally speaking, infringements can be justified when they have a non-arbitrary basis in the law and are necessary for and proportionate to a legitimate aim, such as the protection of the economic well-being of the country; the prevention of crime and disorder; or the protection of health, morals, or the rights and freedoms of others.^108^ For example, some types of nonconsensual medical interventions, such as taking blood for DNA analysis, infringe the right to private life but can be justified when deemed necessary for obtaining evidence of the person’s involvement in the commission of a criminal offense.^109^ Likewise, non-consensual medical treatment, which typically infringes the right to bodily and/or mental integrity, can be permissible in case of ‘medical necessity’.^110^ Hence, compelling claims about the potential overinclusiveness of the human right to mental integrity and the accompanied risk of overregulation cannot be made by merely considering the right’s scope. They should also take account of the right’s permissible limitations, that is, of the ultimate balancing of competing interests that the right allows for.

Still, although qualified rights, such as the right to privacy and bodily integrity, are generally broadly construed in human rights law, opponents of the Significant Mental Interference View may provide reasons to prefer a more specified understanding of the right to *mental* integrity. For example, it could be argued that the Significant Mental Interference View leaves much discretion to (domestic) judges in determining the boundaries of the human rights protection of the mind, as they need to decide on alleged violations of the right to mental integrity based on two open, discretionary tests: (i) whether a mental interference is ‘significant’ and, if so, (ii) whether the interference can be ‘justified’. However, when something as important as accessing and altering persons’ inner mental states is at stake, we should plausibly want a less discretionary test, so it may be argued.^111^

First, it may be true that regarding some very severe mental interferences, less discretion is warranted. In the multi-layered framework of human rights, such severe interferences can be covered by more specific and strict *absolute* rights and prohibitions, such as the prohibition of torture, inhuman, cruel, and degrading treatment; the right to freedom of thought; and the right to freedom of opinion.^112^ Such absolute rights leave less discretion to judges compared to qualified rights. Second, as far as the interest in mental integrity is protected by qualified human rights, the test of ‘justification’ will, indeed, need further guidance. Which factors should be taken into account by (domestic) judges when balancing the competing interests at stake? The next section explores this question.

## IV. EXPLORING PERMISSIBLE LIMITATIONS

Inducing mental changes in others is not unique to neurotechnology. We do so all the time, through a variety of means, ranging from having conversations with others, tweeting opinions and criticism, publishing papers, enforcing compelled education on children, and administering psychopharmaceuticals to psychiatric patients. When not consented to, some such mental influences could infringe a right against significant mental interference. However, not all such infringements are supposedly impermissible. There can be good reasons for bringing about mental changes in persons in different contexts. One could think of educating children and treating patients in forensic psychiatry. But how should human rights law distinguish permissible from impermissible interferences with mental integrity? How should judges and lawmakers determine whether an infringement of the right to mental integrity is either justified or whether it constitutes a violation?

Answering these questions is essential to clarifying the implications of the right mental integrity vis-à-vis different types of mental influencing, including the alteration of mental phenomena by neurotechnology. As Bublitz rightly points out:

The key challenge for the construction of the right to mental integrity lies in developing a taxonomy that sorts the many examples of everyday and undoubtedly permissible social interactions adversely affecting minds of others (causing disappointment, anger, anxiety, etc.) from the supposedly impermissible ones. The legal criteria for this distinction have not been established.^113^

Developing such a taxonomy exceeds the scope of this paper, but let’s explore the following question here: if one would construct the human right to mental integrity as a right against significant mental interference, what types of infringements could then plausibly violate this right?

The answer to this question depends, first, on whether the right to mental integrity is either absolute or qualified. In analogy with the right to bodily integrity—and in accordance with established human rights law—I here assume that a human right to mental integrity is typically a qualified right,^114^ inherent in Article 8 ECHR and 17 ICCPR.^115^

Whether an infringement of the qualified right to mental integrity would constitute a *violation*—and, thus, whether a mental interference is plausibly impermissible—depends, secondly, on an ultimate balancing of competing interests, which, in turn, will largely depend on the facts and circumstances of the individual case. Still, some aspects of this balancing act lend themselves for discussion at a more general level, regardless of the specificities of individual cases. One of them concerns the question of which features of a mental interference are plausibly significant to this balancing of interests. And in what ways? In the remainder of this paper, I explore the potential relevance of three descriptive features of certain types of mental interference, which have been considered normatively relevant in the scholarly literature on the right to mental integrity (though often in different ways). These are: (i) the direct nature of the interference, (ii) its harmful effects, and (iii) the intention of the interferer.

### IV.A. Directness and Harm

The previous sections argued that neither the direct–indirect distinction nor the inducement of physical or psychological harm is a persuasive factor for delimitating the scope of the right to mental integrity. Rather, I suggested that the third construction of the right—as a right against mental interference that attains a certain level seriousness—may fit best within the established framework of human rights. Meanwhile, the directness of a mental interference and its harmful effects can still be relevant to the balancing of interests when an infringement of the right has been determined.

In the law, it is commonly accepted that interferences with a person’s *body* can infringe the right to bodily integrity to different degrees, that is, with different severities. For example, non-consensually inserting a needle in a person’s body will less severely infringe the right compared to non-consensually performing surgery. This is relevant to the ultimate balancing of interests because, in general, the more *severe* the infringement, the more substantial the reasons must be to justify such an infringement. As Gerards explains, the ECtHR ‘has found that stronger and more weighty reasons are needed if an interference is very intrusive or onerous in nature’.^116^ In the same vein, Christoffersen writes that ‘[t]he greater intensity of an interference increases, *ceteris paribus*, the demand for weighty counter-weighing considerations’.^117^ Conversely, less severe interferences require less weighty reasons to be justified.

This plausibly also applies to interference with mental integrity. And this is where the direct–indirect distinction and the infliction of harm become legally relevant. If the primary aim of a right to mental integrity is to protect a person’s *autonomy* over their mental states and processes,^118^ then it is plausible that the less mental *control* an intervention leaves to the targeted person, the more severely the right to mental integrity will be infringed. If we assume that, generally, direct interventions leave less control over mental states than indirect interventions, this implies that, in general, stronger reasons are needed to justify infringements through direct interventions compared to indirect interventions. As such, Bublitz is right when submitting that interventions that undermine or bypass mental control are ‘particularly troublesome’—as they will be harder to justify.

The same *mutatis mutandis* applies to the infliction of psychological harm. When an infringement of the right to mental integrity entails additional psychological injury or mental suffering, for example, by causing anxiety, distress, loss of memory, anger, or depressive feelings, then this will plausibly increase the severity of the rights infringement. Therefore, such infringements require more substantial reasons for potential justification. The relevance of causing *physical* harm is less clear, however, as it is typically the right to *bodily* integrity that accounts for the wrongfulness of inducing detrimental physical effects in others.

### IV.B. Intentionality

Another relevant factor, some might argue, relates to the intentionality of a mental interference. The normative significance of intentional vs. unintentional interference with a person’s mental integrity has recently been considered in debates on the permissibility of administering neurointerventions to convicted persons in order to reduce their risk of recidivism (eg by increasing emphatic abilities and reducing aggressiveness with non-invasive brain stimulation^119^).^120^

The argument, then, goes like this: neurointerventions aim to alter a person’s mental states and processes and therefore affect their mental integrity. Meanwhile, traditional forms of criminal sanctioning, such as incarceration, can equally affect mental integrity, for example, by being detrimental to the person’s mental health.^121^ Still, the latter is not typically considered morally objectionable on the basis of infringing a right to mental integrity. Therefore, prohibiting neurointerventions by appealing to a right to mental integrity would be inconsistent and seems unpersuasive.^122^ However, unlike incarceration, the mental effects induced by neurointerventions are actually intended, while those stemming from incarceration are ‘merely’ foreseeable side-effects. Therefore, the former is more objectionable compared to the latter, leaving space for ruling out neurointerventions based on a right to metal integrity. Put differently: in the context of a right to mental integrity, mental alterations that are a result of *unintended side-effects* are morally less objectionable than *intended* mental changes of similar intensity.

Among others, Birks and Buyx have defended such a claim, arguing that although both neurointerventions and incarceration may involve equal interference with a person’s mental states, harming the person to the same extent and in the same respect, ‘it does not follow that the harm-doing is morally equivalent. There could be a difference in terms of intention between some of the harm-doings caused’.^123^ If intentional harm is harder to justify than unintentional but merely foreseen harm, Birks and Buyx argue, ‘then this could account for the view that neurointerventions are morally objectionable in one respect that incarceration is not’.^124^ In the same vein, Shaw suggests that ‘intended effects and side effects may lie on a spectrum, and the closer the mental-integrity-undermining effects of a punishment or neurointervention fall toward the intended end of the spectrum, the harder the punishment or neurointervention may be to justify’.^125^

The intuition that unintended mental interferences are less morally objectionable than intended interferences may be appealing.^126^ Meanwhile, the *legal* significance of this distinction in the context of the human right to mental integrity plausibly depends on how one understands this right. Birks and Buyx understand the ‘interest in mental integrity’ as ‘a person’s interest in not having at least some of his mental states *intentionally* altered by others in certain ways’.^127^ Likewise, Shaw holds that the moral right to mental integrity ‘can be infringed by *intentionally* interfering with a person’s mental states through nonrational means’.^128^

Indeed, *if* the right to mental integrity is understood as a right against some forms of *intentional* mental interference, the degree of intentionality is plausibly relevant for determining the severity of a rights infringement, which, in turn, affects the balancing of interests and, therefore, potential justification of the infringement. This could mean that *knowingly* causing mental changes in others, like by sending a person to prison or by selling neurodevices that can have adverse psychological side-effects in their users, would less severely infringe the right to mental integrity than *purposely* inducing mental change of similar intensity, for example, by compelling a person to psychopharmaceuticals or brain stimulation to prevent future harm or by manipulating a person’s preferences and intentions for political or economic gain.

However, if one constructs the right to mental integrity without the conditional requirement of intentionality—such as a right against ‘significant mental interference’—the legal relevance of different degrees of intentionality seems less straightforward,^129^ also because, typically, to be justified, infringements of the right to mental integrity *need to be* intentional: they must pursue a ‘legitimate aim’ and be proportionate to that aim, such as the prevention of crime or the protection of health and the moral well-being of others.^130^ Note, finally, that adopting an ‘intentionality-requirement’ for constructing the right’s scope could result in reduced human rights protection against interventions that have serious side-effects on people’s minds. For example, such could imply that serious unintended side-effects of mandatory anti-libidinal interventions in forensic psychiatry, such as depressive symptoms, lethargy, mental disorders, emotional disturbances, or anxiety, will not be covered by the protective scope of the right to mental integrity, just because they were not intended.^131^

## V. CONCLUDING REMARKS

Modern (neuro)technologies have been considered to pose an increasing threat to people’s autonomy over their mental states and processes. One way to guarantee adequate legal protection against new and emerging forms of unsolicited mental interference is by specifying human rights that protect mental autonomy. This includes specifying the right to mental integrity. This right is currently underdeveloped—both in theory and practice. The Human Rights Council Advisory Committee has stressed the importance of clarifying the right to mental integrity and its potential implications for neurotechnology. To this end, the present paper considered three possible ways of constructing the scope of the right to mental integrity in human rights law. It was argued that the Mental Control View and the Direct Harmful Interference View appear, generally, of little help (if any) to clarify and delineate the protective scope of the right. Rather, this paper tentatively proposes to construct the scope of the right to mental integrity along the lines of the Significant Mental Interference View. On this view, the *directness* of a mental interference and the psychological *harm* it entails are plausibly relevant factors to the possible justification of rights infringements and, therefore, to the (im)permissibility of different types of mental interference.

## Footnotes

1 For overview and discussion of these neurotechnologies, see N. Farahany, The  Battle for  Your  Brain: Defending the  Right to  Think  Freely in the  Age of  Neurotechnology (2023). For recent policy initiatives see, for example, UNHRC, Neurotechnology and  Human  Rights, A/HRC/51/L.3 (29 Sept. 2022); UNESCO, First draft of the  Recommendation on the  Ethics of  Neurotechnology (2024); J.C. Bublitz & S. Ligthart, *The New Regulation of Non-Medical Neurotechnologies in the European Union*, 11 JLB lsae021 (2024).

2 J.C. Bublitz & R. Merkel, *Crimes Against Minds: On Mental Manipulations, Harms and a Human Right to Mental Self-Determination*, 8 Crim Law Phil 51 (2014); M. Ienca & R. Andorno, *Towards new human rights in the age of neuroscience and neurotechnology*, 13 LSSP (2017).

3 Advisory Committee, UNHRC, Impact, Opportunities and Challenges of Neurotechnology With Regard to the Promotion and Protection of All Human Rights, A/HRC/AC/31/CRP.1 (8 August 2024), at 78. Cf. M. Ienca, Common Human Rights Challenges Raised by Different Applications of Neurotechnologies in the Biomedical Field, Report commissioned by the Committee on Bioethics of the Council of Europe (2021).

4 See, for example, Ienca & Andorno *supra* note 2; J.C. Bublitz, *Neurotechnologies and human rights: restating and reaffirming the multi-layered protection of the person,* 28 IJHR 782 (2024).

5 Advisory Committee, *supra* note 3, at 63.

6 T. Istace, *Establishing Neurorights: New Rights versus Derived Rights,* 17 J. Hum. Rights  Pract. 121 (2025).

7 S. Ligthart et al., *Minding Rights: Mapping Ethical and Legal Foundations of ‘Neurorights’*, Camb C Healthc  Ethic 32 (2023).

8 Ienca & Andorno, *supra* note 2.

9 Bublitz, *supra* note 4. See also T. Istace, *Human rights law: an incomplete but flexible framework to protect the human mind against neurotechnological intrusions*, 16 Law  Innov. Technol 309 (2024); S. Michalowski, *Critical Reflections on the Need for a Right to Mental Self-Determination*, in The  Cambridge  Handbook of  New  Human  Rights: Recognition, Novelty, Rhetoric (A. von Arnauld, K. von der Decken & M. Susi eds., 2020).

10
*Supra* note 3.

11 Istace, *supra* note 9. For discussion on the right’s theoretical foundations, see T. Douglas & L. Forsberg, L., *Three Rationales for a Legal Right to Mental Integrity*, in Neurolaw: Advances in  Neuroscience, Justice, and  Security (S. Ligthart et al. eds, 2021).

12 Cf. T. Istace, *Exploring the Essence of the Freedom of Thought – A Normative Framework for Identifying Undue Mind Interventions*, 18 Neuroethics 10 (2025).

13 Advisory Committee, *supra* note 3, at 83 (together with the right to freedom of thought).

14 Here, I set aside the question of whether human rights are the most appropriate tool to effectively protect people’s mental integrity, for example, compared to domestic (criminal, civil, administrative) laws or soft-law regulations.

15 Some philosophers take a moral right to mental integrity to include the protection of mental privacy. See, eg A. Lavazza, *Freedom of Thought and Mental Integrity: The Moral Requirements for Any Neural Prosthesis*, 12(82) Front. Neurosci. 1 (2018). In accordance with the dominant view in the literature, this paper approaches the human rights protection of mental integrity as a freestanding and separate issue from the protection of mental privacy.

16 See eg *supra* note 2.

17 See eg S. Ligthart, E. Dore-Horgan & G. Meynen, Minds, Freedoms and  Rights (in press).

18 And on some interpretations, the mental effects of a brain intervention could be relevant to evaluations concerning the right to bodily integrity: V. Tesink et al., *Neurointerventions in Criminal Justice: On the Scope of the Moral Right to Bodily Integrity*, 6 Neuroethics 2023, 1 (2023).

19 Bublitz, *supra* note 4 at 7. Consider, for example, non-invasive brain stimulation with tDCS or TMS.

20
*Id*.; N. Craig, *Incarceration, Direct Brain Intervention, and the Right to Mental Integrity – a Reply to Thomas Douglas*, 9 Neuroethics 107 (2016); E. Shaw, *Neuroscience, Criminal Sentencing, and Human Rights*, 63 Wm. & Mary L. Rev. 1409 (2022); J. Ryberg, Neurointerventions, Crime, and  Punishment (2020), at 83.

21 See, eg ECtHR (GC) 11 July 2006, 54810/00 (*Jalloh/Germany*), par. 82.

22 M. Nowak/W. Schabas, U.N. International  Covenant on  Civil and  Political  Rights: Nowak’s CCPR Commentary (2019), at. 170. Cf. P.M. Taylor, A Commentary on the  International  Covenant on  Civil and  Political  Rights (2020), at 199.

23 CCPR General comment No. 35: Article 9, par. 3.

24 Nowak/Schabas, *supra* note 22, at 469; K. de Vries, *Right to Respect for Private and Family Life*, in Theory and  Practice of the  European  Convention on  Human  Rights (P. van Dijk et al. eds., 2018), at 690–691.

25 ECtHR 11 May 2023, 11148/18 (*Bojar/Poland*), par. 12. See also: ECtHR 12 Oct. 2006, 3178/03 (*Mayeka and Kaniki Mitunga/Belgium*), par. 83.

26 De Vries, *supra* note 24.

27 ECtHR 6 Feb. 2001, 44599/98 (*Bensaid/UK*), par. 47.

28 ECtHR 24 July 2012, 41526/10 (*Đorđević/Croatia*), par. 97–98.

29 ECtHR 30 Nov. 2010, 2660/03 (*Hajduová/Slovakia*), par. 49.

30 ECtHR 28 Oct. 2014, 531/06 (*Ion Cârstea/Romania*) par. 38; ECtHR 21 Nov. 2013, 16882/03 (*Putistin/Ukraine*), par. 32.

31 ECtHR 6 Feb. 2001, 44599/98 (*Bensaid/UK*), par. 46.

32 De Vries, *supra* note 24 p. 691. See also ECtHR 25 Mar. 1993, 13134/87 (*Costello-Roberts/UK*).

33 D.J. Harris et al., Harris, O’Boyle, and  Warbrick: Law of the  European  Convention on  Human  Rights (2023), p. 525.

34 ECtHR 16 June 2005, 61603/00 (*Storck/*Germany), par. 143.

35 ECtHR 15 May 2018, 41079/16 (*Caruana/Malta*), par. 41. Also

36 Harris et al., *supra* note 33, at 528. See also De Vries, *supra* note 24, p. 693.

37 Bublitz & Merkel, *supra* note 2; Michalowski, *supra* note 9; Istace, *supra* note 9. In some domestic legal systems, the right has been extensively debated, though. See, eg S. Ruiz et al., *Neurorights in the Constitution: from neurotechnology to ethics and politics*, Philos  Trans R Soc  Lond B Biol  Sci., DOI: 10.1098/rstb.2023.0098 (2024).

38 Craig, *supra* note 20; Lavazza, *supra* note 15; Shaw, *supra* note 20; H. Zohny, et al., *The Mystery of Mental Integrity: Clarifying Its Relevance to Neurotechnologies*, 18 Neuroethics 20 (2023); V. Tesink et al., *Right to mental integrity and neurotechnologies: implications of the extended mind thesis*, 50 JME 656 (2024); W. Ratoff, *The right to mental autonomy: It’s nature and scope*, 27 JESP 257 (2024); G. Cassinadri, *The Right to Mental Integrity: Multidimensional, Multilayered and Extended*, 18 Neuroethics 16 (2025).

39 Cf. Ligthart et al., *supra* note 7.

40 That is, as part of the qualified right to privacy and respect for private life, which seem to provide most space for carving out a general right to mental integrity, due to their qualified nature and the absence of a clear injury-requirement.

41 Lavazza, *supra* note 15, at 4 (emphasis added).

42 E. Hildt, *A Conceptual Approach to the Right to Mental Integrity*, in Protecting the  Mind (P. López-Silva & L. Valera, eds., 2022), at 88 (emphasis added).

43 Advisory Committee, UNHRC, Draft Report on Impact, Opportunities and Challenges of Neurotechnology with Regard to the Promotion and Protection of All Human Rights, A/HRC/AC/31/CRP.1 (12 Feb. 2024), par 35 (emphasis added).

44 Advisory Committee, *supra* note 3, at 27 (emphasis added).

45 Bublitz & Merkel, *supra* note 2. Also J.C. Bublitz, *Why means matter*, in Neurointerventions and the  Law: Regulating  Human  Mental  Capacity (N.A. Vincent, T. Nadelhoffer & A. McCay, eds., 2020), at 69. In the same direction, see Istace, *supra* note 9, at 337.

46 Bublitz & Merkel, *supra* note 2, at 68.

47 Bublitz & Merkel, *supra* note 2, at 62.

48 Douglas & Forsberg, *supra* note 11, at 191.

49 For discussions over moral justifications of a right to mental integrity, see Douglas & Forsberg, *supra* note 11; A. Lavazza & R. Giorgi, *Philosophical foundations of the right to mental integrity*, 16 Neuroethics 10 (2023); T. Douglas, *An Intuitive, Abductive Argument for a Right Against Mental Interference*, The  Journal of  Ethics (2024, doi.org/10.1007/s10892-024-09476-7).

50 Zohny et al., *supra* note 38, at 19.

51
*Id.,* at 20.

52 Bublitz & Merkel, *supra* note 2, at 65.

53 Zohny et al., *supra* note 38, at 20.

54 Zohny et al., *supra* note 38, at 20 (emphasis added).

55 Eg Bublitz, *supra* note 4; Douglas & Forsberg, *supra* note 11; Ligthart et al., *supra* note 7; Lavazza & Giorgi, *supra* note 49; Bublitz & Merkel, *supra* note 2.

56 Douglas ,*supra* note 49; Ratof,f *supra* note 38, at 258; Ryberg, *supra* note 20, at 83.

57 Ienca & Andorno, *supra* note 2, at 18.

58
*Id.* See also Cassinadri *supra* note 38, who aims to expand and refine Ienca and Andorno’s characterization of the right to mental integrity.

59 Zohny et al., *supra* note 38; Bublitz, *supra* note 45; T. Douglas, *Neural and Environmental Modulation of Motivation: What’s the Moral Difference?*, in Treatment for  Crime (D. Birks & T. Douglas, eds., 2018).

60 Zohny et al., *supra* note 38.

61 Bublitz & Merkel, *supra* note 2. See also Bublitz, *supra* note 45.

62 Bublitz, *supra* note 45; Bublitz & Merkel, *supra* note 2; Zohny et al., *supra* note 38, at 7.

63 Bublitz & Merkel, *supra* note 2; Bublitz, *supra* note 45.

64 See, for example, P. Zuk, *Mental integrity, autonomy, and fundamental interests*, 50 JME 676 (2024).

65 Ratoff, *supra* note 38, at 282.

66 Zohny, et al. *supra* note 38, at 20.

67 Bublitz, *supra* note 45, at 70.

68
*Id*.

69 Douglas, *supra* note 59.

70 N. Levy, *Cognitive Enhancement: Defending the Parity Principle,* in Neurointerventions and the law (N.A. Vincent, T. Nadelhoffer & A. McCay eds., 2020). See also N. Levy, Neuroethics: Challenges for the 21st  Century (2007).

71 Ryberg, *supra* note 20, at 91.

72 ECtHR 30 Nov. 2010, 2660/03 (*Hajduová/Slovakia*), par. 49; ECtHR 24 July 2012, 41526/10 (*Đorđević/Croatia*), par. 97.

73 ECtHR 28 Oct. 2014, 531/06 (*Ion Cârstea/Romania*) par. 38.

74 Nowak/Schabas, *supra* note 22, at 469.

75 EU Network of Independent Experts on Fundamental Rights, Commentary of the  Charter of  Fundamental  Rights of the  European  Union (2006).

76 Which is a freestanding right next to the right to freedom of conscience and religion, guaranteed by Article 18 ICCPR and 9 ECHR. Unlike the right to mental integrity, the right to freedom of thought is often considered an absolute right. Meanwhile, its scope is plausibly narrower, as it only applies to mental properties qualifying as ‘thoughts’. For recent discussion of the right's absolute nature, see S. Ligthart, *Reconsidering the Absolute Nature of the Right to Freedom of thought*, Human Rights Law Review 2025, 10.1093/jlb/lsaf010.

77 Nowak/Schabas ,*supra* note 22, at 503; B. Vermeulen & M. Roosmalen, *Freedom of Thought, Conscience and Religion*, in Theory and  Practice of the  European  Convention on  Human  Rights (P. van Dijk et al. eds., 2018)738.

78 UN Special Rapporteur on the Freedom of religion or belief, 5 Oct 2021, A/76/380, par. 35.

79 See also Istace, *supra* note 12; S. Alegre, Freedom to Think (2022); McCarthy-Jones, Freethinking (2024).

80 See, eg Ienca and Adorno’s proposal, which would limit the right’s scope to actions involving ‘the direct access to and manipulation of neural signalling’.

81 Zohny et al., *supra* note 38, at 4.

82
*Id.* (original emphasis).

83
*Id.* They also refer to mental influence through the news, advertisements, and interaction with friends and strangers, all being capable to harmfully interfere with our mental states.

84 Bublitz, *supra* note 4, at 7.

85 Insofar as they do not cause mental distress or otherwise adversely affect the person’s well-being. Again, this would depend on how one defines harm, see, eg Istace *supra* note 9, at 338, maintaining that, in the context of the right to mental integrity, ‘[t]he undermining of mental autonomy or self-determination could be convincingly labelled as mental harm’. See also Cassinadri *supra* note 38, proposing to specify the harm-requirement to phenomenological mental harms (like mental pain, suffering, and distress) and functional mental harm (like induced dysfunctions or incapacities).

86 Harris et al., *supra* note 33, at 243; B. Vermeulen & H. Battjes, *Prohibition of torture and other inhuman or degrading treatment or punishment*, in Theory and  Practice of the  European  Convention on  Human  Rights (P. van Dijk et al. eds., 2018), at 387, 384.

87 JnnC Bublitz, *The Nascent Right to Psychological Integrity and Mental Self-Determination*, in The  Cambridge  Handbook of  New  Human  Rights: Recognition, Novelty, Rhetoric (A. von Arnauld, K. von der Decken & M. Susi, eds. 2020), at 396.

88 S.E. Biber & M. Capasso, *The Right to Mental Integrity in the Age of Artificial Intelligence: Cognitive Human Enhancement Technologies*, in Law and  Artificial  Intelligence (B. Custers and E. Fosch-Villaronga, eds., 2022), at 511.

89 Cf. Zohny et al., *supra* note 38, at 4; Zuk, *supra* note 65, at 3; Bublitz, *supra* note 4, at 7; Ligthart et al., *supra* note 7, at 6.

90 Douglas & Forsberg, *supra* note 11, at 182. See also Douglas, *supra* note 49; Ligthart et al., *supra* note 7, at 6.

91 Douglas & Forsberg, *supra* note 11, at186.

92 Douglas, *supra* note 59, at 6, adding in footnote 16 that ‘we might suppose that this will depend on how many mental states or parts are interfered with, on the centrality and/or importance of those mental states or parts, and on the magnitude of any interferences with them’.

93 Bublitz, *supra* note 4, at 7.

94 Cf. Douglas, *supra* note 49, at footnote 16.

95 Bublitz, *supra* note 4, at 7.

96 Douglas ,*supra* note 49; Bublitz, *supra* note 4. As Bublitz writes, ‘all neurointerventions with *non-trivial* effects fall under the [right’s] scope’ (at 7, emphasis added).

97 ECtHR 6 Feb. 2001, 44,599/98 (*Bensaid/UK*), par. 46.

98 Harris et al., *supra* note 33, at 525.

99 Ratof,f *supra* note 38, at 264; Cassinadri, *supra* note 38, at 3; J. Blumenthal-Barby, & P. Ubel, *Neurorights in question: rethinking the concept of mental integrity*, 50 JME 670. Cf. Ryberg, *supra* note 20, at 84–92.

100 Eg ECtHR (GC) 25 September 2018, 76639/11 (*Denisov/Ukraine*), § 95. For the interpretation of ‘private life’, the ECtHR refers to another unspecified principle underlying the interpretation of Article 8 ECHR, that of ‘personal autonomy’: ECtHR (GC) 5 September 2017, 61,496/08 (*Bărbulescu/Romania*), § 70.

101 CCPR General Comment No. 20: Article 7, par. 4.

102 Harris et al., *supra* note 33, at 244.

103 Though there could be good reasons for applying (some) human rights to private companies, too. Cf. United Nations Guiding Principles on Business and Human Rights.

104 Note that the mechanisms used by private compagnies to obtain consent for monitoring, collecting, and using information about mental states and processes, and possibly altering them, can sometimes be opaque.

105 Still, some everyday interferences might infringe the right, for example, if we are repeatedly exposed to pop-up ads to which we did not consent.

106 De Vries, *supra* note 24, at 667.

107 K. Möller, The  Global  Model of  Constitutional  Rights (2012), at 4.

108 Nowak/Schabas, *supra* note 22, at 463–46; L. Lavrysen, *System of Restrictions*, in Theory and  Practice of the  European  Convention on  Human  Rights (P. van Dijk et al. eds. 2018).

109 ECtHR 15 May 2018, 41,079/16 (*Caruana/Malta*), par. 31; ECtHR (GC) 11 July 2006, 54810/00 (*Jalloh/Germany*), par. 70

110 Harris et al., *supra* note 33, at 528; W. Buelens, C. Herijgers & S. Illegems, *The View of the European Court of Human Rights on Competent Patients’ Right of Informed Consent* 23 EJHL 481 (2016).

111 I thank an anonymous reviewer for raising this point.

112 Bublitz, *supra* note 4. Regarding the absolute nature of the right to freedom of thought, see S. Ligthart, *Reconsidering the Absolute Nature of the Right to Freedom of Thought*, HRLR 2025 (in press).

113 Bublitz, *supra* note 4, at 7. See also Istace, *supra* note 9, at 338. Cf. Nowak/Schabas, *supra* note 22, at 503.

114 Cf. Ryberg, *supra* note 20, at 92; Craig, *supra* note 20; G. Mendlow, *Why Is It Wrong To Punish Thought?* 127 Yale L.J. 2342 (2018).

115 Note that some types of mental inference could engage absolute protection in some cases, for example, from the prohibition of ill-treatment or the right to freedom of thought.

116 J. Gerards, General  Principles of the  European  Convention on  Human  Rights (2023), at 355.

117 J. Christoffersen, Fair  Balance: Proportionality, Subsidiarity and  Primarity in the  European  Convention on  Human  Rights (2009), at 208.

118 Which is plausibly the case when the right is constructed within Article 17 ICCPR and 8 ECHR. For example, personal autonomy is an important principle underlying the interpretation of Article 8 ECHR: ECtHR (GC) 5 Sept. 2017, 61496/08 (*Bărbulescu/Romania*), § 70.

119 See, eg C.S. Sergiou et al., *Transcranial Direct Current Stimulation Targeting the Ventromedial Prefrontal Cortex Reduces Reactive Aggression and Modulates Electrophysiological Responses in a Forensic Population*, 7 Biol  Psychiat: CNNI 95 (2022). See also R. Knehans et al., *Modulating Behavioural and Self-Reported Aggression with Non-Invasive Brain Stimulation: A Literature Review*, 12 Brain  Sci. 200 (2022).

120 Shaw, *supra* note 20.

121 J. Schnittker, *The Psychological Dimensions and the Social Consequences of Incarceration*, 651 Ann. Am. Acad. Political  Soc. Sci. 122 (2014).

122 Ryberg, *supra* note 20, at 86.

123 D. Birks & A. Buyx, *Punishing Intentions and Neurointerventions*, 9 AJOB Neuroscience 133 (2018), at 134.

124
*Id*.

125 Shaw, *supra* note 20.

126 For philosophical discussion of the moral significance of intentionally versus foreseeably causing harm, see, for example, M. Moschella, *Contextualizing, Clarifying, and Defending the Doctrine of Double Effect*, 26 JESP 297 (2023).

127 Birks & Buyx, *supra* note 123, at 136 (emphasis added).

128 Shaw, *supra* note 20, p. 1418 (emphasis added). Cf. Istace, *supra* note 12.

129 Consider, for example, the non-intentional but merely foreseeable mental suffering caused by the execution of an irreducible life prison sentence, which can qualify as inhuman or degrading treatment in violation of Article 3 ECHR, despite the lack of intentionality: ECtHR (GC) 26 Apr. 2016, 10511/10 (*Murray/The Netherlands*); ECtHR (GC) 9 July 2013, 66069/09, 130/10 and 3896/10 (*Vinter et al./UK*).

130 As follows from Article 8(2) ECHR.

131 Cf. L. Forsberg, *Anti-libidinal Interventions and Human Rights*, 21 HRLV 384 (2021); Istace, *supra* note 12 at 15.

